# Effects of students’ perception of teachers’ ethnic-racial socialization on students’ ethnic identity and mental health in rural China’s schools

**DOI:** 10.3389/fpsyg.2023.1275367

**Published:** 2024-02-15

**Authors:** Angel Hor Yan Lai, Jason K. H. Lam, Hong Yao, Elaine Tsui, Cynthia Leung

**Affiliations:** ^1^Hong Kong Polytechnic University, Kowloon, Hong Kong SAR, China; ^2^Minzu University of China, Beijing, China; ^3^School of Continuing Education, Hong Kong Baptist University, Kowloon, Hong Kong SAR, China

**Keywords:** ethnic-racial socialization, ethnic identity, ethnic minority, stress, mental health, China, teacher

## Abstract

**Introduction:**

Using students in the Liangshan Yi autonomous prefectures of southwestern China (*n* = 585; 13–15 years old), we examined (i) the effects of students’ perception of their teachers’ ethnic-racial socialization on their ethnic identity and mental health outcomes of depressive and stress symptoms; (ii) the effects of students’ ethnic identity on their depressive and stress symptoms; (iii) the differential associations among these factors in Yi ethnic minority versus Han cultural majority students.

**Methods:**

We conducted a cross-sectional survey and used multistage sampling to collect the information. Chinese-validated standardized measures were used: the Patient Health Questionaires-9, Children’s Revised Impact of Event Scale-8, Multigroup Ethnic Identity Measure, Cultural Socialization Scale, and Teachers’ Attitude on Adoption of Cultural Diversity Scale. Multigroup confirmatory factor analysis and multigroup structural equation modeling were employed.

**Results:**

Comparing the findings in Yi and Han students, their perception of teachers’ ethnic-racial socialization had dissimilar effects on their ethnic identity and mental health outcomes. Three key findings comparing the differences between Yi and Han students were as follows: (i) students’ perception of their teachers’ multicultural socialization practices positively affected the ethnic identity of both Yi and Han young people; however, their perception of their teachers’ socializing them to their own cultures did not exert any effect; (ii) students’ perception of teachers’ multicultural socialization practices had different mental health effects on Yi versus Han students; and (iii) ethnic identity affected the mental health of Yi ethnic minority students only.

**Conclusion:**

The findings underscore the importance of teachers’ multicultural socialization in the ethnic identity development of both Yi ethnic minority and Han majority students. Ethnic identity serves as a linking variable bridging perceived teachers’ multicultural socialization practices and mental health in Yi ethnic minority students but not among the Han cultural majority youths. Research, practice, and policy implications relevant to the global context are also discussed.

## Introduction

Ethnic identity constitutes an integral part of young people’s social identity, particularly as adolescents build their unique sense of self (Umaña-Taylor, 2023). For ethnic-racial minority individuals, ethnic identity protects them from racial discrimination and facilitates them to understand what their cultural lineage means to them (Tajfel and Turner, 1986; Phinney and Ong, 2007), which, in turn, promotes their mental health (e.g., Rivas-Drake et al., 2014; Umana-Taylor et al., 2014). Supportive ethnic-racial socialization in school settings is a proven source of strong ethnic identity and positive psychological health in ethnic-racial minority young people in Western literature (e.g., Byrd, 2019; Saleem and Byrd, 2021). Research conducted by the authors of this article among Asian students also showed that through learning about and discussing their own cultures in relation to larger society and issues on racial equality and justice at their schools, which is, an immediate social context that young people are nested in, students are given the opportunity to resolve ethnic uncertainties and leverage their ethnicity to develop their sense of life purpose (e.g., Lai et al., 2023; Teyra et al., 2023). While scholars have been focusing on the study of ethnic identity in ethnic-minority young people, there is also a growing discussion on the importance of ethnic identity for the cultural majority. For the general population, ethnic identity facilitates them to understand their role as their cultural oppressor in history, realize their racial privileges and responsibilities in society, which will contribute to their positive self-development and multicultural integration and equality in broader society (Moffitt and Rogers, 2022; Umaña-Taylor, 2023).

On the other hand, literature on school ethnic-racial socialization and ethnic identity in non-Western settings remains scant especially research on their ethnic-racial socialization experiences, ethnic identity development, and psychological well-being (Umaña-Taylor, 2023). In Asia, the term “ethnic minority” refers to both indigenous peoples and immigrants, with the definition varying across regions. Taking the example of China, the largest country in East Asia, ethnic minority often refers to indigenous peoples. In China, studies on ethnic minorities sought to understand their physical health, socio-economic outcomes, and issues related to community development (e.g., Huang et al., 2017; Postiglione, 2017; Gustafsson et al., 2022), with a growing scholarship on ethnic or national identity and psychological health, including studies conducted by the authors of this article (e.g., Lai et al., 2017, 2019, 2022a,b; Guan et al., 2022a). Existing research has also explored the experiences of indigenous peoples in Japan and Taiwan, most often about their history, cultures, and issues related to racial injustice but not their ethnic identity (e.g., Weiner, 2009; Huang et al., 2021; Lai and Teyra, 2023). To promote cross-cultural scholarship and advance our knowledge, additional research is needed especially in areas relating the cultural identity and well-being of multi-cultural community in non-Western settings.

Using youths in the Liangshan Yi Autonomous Prefecture (Liangshan) in southwestern rural China, this study examined the effects of students’ perception of teacher ethnic-racial socialization on their ethnic identity and mental health, including depressive and stress symptoms. Participants were students of Yi and Han cultural origin, the two major ethnic groups in Liangshan. Han people are the ethnic majority in China, comprising over 90% of the country’s total population (National Bureau of Statistics of China, 2021). While the Yi are an ethnic minority at the country level, they are the indigenous people in Liangshan, constituting over 50% of the region’s total population. The rest of the population in Liangshan is dominated by the Han. The ethnic-majority versus -minority dynamics in this autonomous administrative division set the stage for an interesting research question: How does school context affect the ethnic identity and mental health of the multicultural youth community in Liangshan? Guided by ethnic identity development theory (Phinney and Ong, 2007) and the school-based ethnic-racial socialization framework (Saleem and Byrd, 2021), this study conceptualized that students’ perception of their teachers’ ethnic-racial socialization practices positively affected students’ ethnic identity, which then influenced those students’ mental health. We also compared the differences in the associations among these variables between Yi and Han students.

### Ethnic identity and mental health

Ethnic identity is the degree to which an individual identifies as a member of an ethnic group (Phinney and Ong, 2007; Umana-Taylor et al., 2014). It comprises two components: ethnic identity commitment and ethnic identity resolution (Phinney and Ong, 2007; Umana-Taylor et al., 2014). Strong ethnic identity commitment is defined as having a positive feeling and a sense of belonging to one’s own cultural community. Ethnic identity resolution refers to individuals having resolved their ethnic uncertainties, attaching personal meaning to their own ethnic group membership, and having a willingness to continue to learn about one’s own culture. The development of ethnic identity is described as having three key stages (Phinney and Ong, 2007), which include: (1) Diffusion/ Foreclosure, when individuals are aware of their ethnic identity, yet, have little understanding or knowledge about their cultural roots; (2) Moratorium, when individuals start contemplating and exploring the relationship between themselves and their ethnic roots, and feeling perplexed about ethnic uncertainties; (3) Achieved identity, when individuals develop a consolidated and strong commitment to their ethnic identity and having resolved their ethnic uncertainties. The formation of ethnic identity is most salient during adolescence, when young people develop an abstract thinking ability to search for their unique selves in relation to their external social world, including their cultural communities (Umaña-Taylor, 2023).

The notion of ethnic identity has been studied in the Asian societies, with qualitative research in Mainland China showing that young people also experienced similar sentiment towards their ethnic identity. In China’s university settings, Zhuang ethnic minority students showed different patterns of ethnic identification as influenced by their personal experiences and China’s cultural policies (Lu and Gu, 2021). These patterns included: receivers, who accepted their ethnic classification but have limited ethnic awareness; constructors, who had strong ethnic awareness and strived to protect their cultural heritage; and utilizers, who saw their ethnicity as their asset of obtaining more resources. These ethnic identifications, to a certain extent, aligned with the stages of ethnic identity development as stated by Phinney and Ong (2007). The receivers echoed with those who were at the diffusion/ foreclosure stage, while constructors seemed to be those who are having a consolidated sense of achieved ethnic identity.

Furthermore, in rural China secondary schools, ethnic minority students displayed various feelings towards their ethnicity depending on their school cultural experiences, which was consistent with findings in the U.S. (e.g., Umaña-Taylor, 2023). Some showed pride and positive feelings towards their ethnic group membership, while others preferred to disengaged from their ethnic communities because of racial discrimination. On the other hand, some students were able to resolve their racial discrimination experiences and pledged to contribute to their ethnic community with the mainstream knowledge they learned at school (Lai et al., 2023). These findings suggested that ethnicity do play a special role within the development of ethnic minority individuals in different socio-cultural settings. Even though specific socio-cultural circumstances might have affected their ethnic identity development differently, the general patterns of ethnic identification, to a certain extent, were manifested similarly across multicultural groups internationally.

The mental and psychological health benefits of strong ethnic identity commitment and resolution have been demonstrated in previous research, for example, lower depressive and anxiety symptoms and better self-esteem, self-efficacy, and academic motivation in multiracial and ethnic-minority young people, in both Western and Asian societies (e.g., Smith and Silva, 2011; Rivas-Drake et al., 2014; Lai et al., 2019; Kuang and Nishikawa, 2021; Long et al., 2021; Lai et al., 2022a,b). However, ethnic identity commitment without resolution is not healthy (Tajfel and Turner, 1986; Phinney, 1996; Umaña-Taylor, 2023). For cultural groups that are treated as inferior, those who are strongly committed to their ethnic groups may feel frustrated (Tajfel and Turner, 1986). For instance, ethnic minority individuals with unresolved questions about their ethnicity tend to show poorer psychological and mental health, such as lower levels of life satisfaction and self-esteem, as seen in the U.S. (Syed et al., 2013), and higher levels of depression and anxiety, as seen in Taiwan (Lai and Teyra, 2023). This study accumulated further knowledge on the association between ethnic identity and mental health by examining the effects of ethnic identity commitment and resolution on depression and stress symptoms in Yi and Han students in Liangshan.

### School ethnic-racial socialization, ethnic identity, and mental health

Ethnic-racial socialization is a key source of ethnic identity and psychological well-being, especially in multicultural communities that were treated as the “inferior” minorities in their societies (Hughes et al., 2006; Saleem and Byrd, 2021). Through social interactions with families, teachers, and friends, these young people develop insights into the meaning of their ethnicity and a deep attachment to their ethnic group membership, which, in turn, impacts their psychological health (Hughes et al., 2006; Saleem and Byrd, 2021; Umaña-Taylor, 2023). This study focused on students’ perceptions of their teachers’ ethnic-racial socialization in schools. In rural China’s educational settings, where most students board at school because of long home–school traveling distances, teachers are their immediate family members and role models. As such, we borrowed the school ethnic-racial socialization framework (Saleem and Byrd, 2021) to conceptualize the pathways from teachers’ ethnic-racial socialization to ethnic identify and mental health.

According to the school ethnic-racial socialization framework (Saleem and Byrd, 2021), five ethnic-racial socialization practices in schools influence students’ ethnic identity and well-being: cultural socialization, multicultural socialization, critical consciousness, mainstream socialization, and colorblind socialization. Cultural and multicultural socialization are supportive practices that focus on learning and respecting one’s own and other people’s cultures. Critical consciousness includes messages confronting racial oppression and injustice. Mainstream and colorblind socialization are messages that encourage cultural assimilation into the mainstream culture. This study focused on the practices of cultural and multicultural socialization, considering the strength-based nature of this research and China’s cultural policy, which emphasizes the promotion of harmony and integration rather than racial justice (Postiglione, 2017).

Cultural socialization is a practice that supports individuals in understanding, appreciating, and affirming their ethnic heritage, traditions, and history (Saleem and Byrd, 2021). School cultural socialization facilitate students in building a meaningful and deep connection to their ethnicities via cultural discussion and participation (Saleem and Byrd, 2021). Cultural socialization was found to be associated with strong ethnic identity in a multi-ethnic-racial group of adolescents in the U.S. (Byrd, 2019; Del Toro and Wang, 2020). However, its effect on educational outcomes was mixed. Byrd (2019) showed that it was not related to school belonging and competence, while Del Toro and Wang (2020) found that it was positively associated with academic performance as mediated by ethnic identity. To add new evidence to the field, this study examined the effects of students’ perception of teachers’ cultural socialization on the ethnic identity and mental health of Yi and Han ethnic youths in rural China.

Multicultural socialization is the practice of teaching about other people’s cultures, respecting cultural diversity, and paying equal attention to individuals from different cultural backgrounds (Saleem and Byrd, 2021). Learning about and respecting other cultures provides an opportunity for students to compare their ethnic experiences with those of out-group cultures and reflect on their ethnic values and practices in relation to the larger society (Saleem and Byrd, 2021). Research on multi-ethnic and racial groups in the U.S. showed that interacting with teachers who embrace cultural diversity promotes students’ ethnic identity and mental health (Byrd and Chavous, 2011; Brown and Chu, 2012). A study of rural China Yi students also found that they developed a meaning and purpose in their lives by pledging to integrate their newly learned mainstream Han practices at school with their traditional cultural values to promote their community well-being (Lai et al., 2023). Based on the literature, this study examined the effects of students’ perception of teachers’ multicultural socialization on the ethnic identity and mental health of Yi and Han ethnic youths. In this research, we defined teachers’ multicultural socialization as the students’ perception of their teachers’ cultural diversity attitudes and their practices of socializing their students with the other dominating culture, that is, the Yi and Han culture in Liangshan context.

### Ethnic identity and ethnic-racial socialization in the cultural majority

Scholars have been advocating for the salience of ethnic identity development in cultural majorities because having a strong ethnic identity increases their cultural awareness and supports them in understanding their privileged status based on race (e.g., Moffitt and Rogers, 2022; Umaña-Taylor, 2023). However, studies showed that most young White people in the U.S., because of their majority status, tend to remain at the initial “colorblind” and “ignorant” stage of ethnic-racial awareness and do not see racial or ethnic inequality as relevant to them (Hardiman and Keehn, 2012; Ison et al., 2023). The findings suggested that ethnic identity carried dissimilar meanings to the mainstream and cultural-minority populations. As ethnic majorities and minorities are exposed to distinct ethnic-racial socialization messages given their dissimilar cultural status and background in their societies, it makes sense that the effects of teachers’ ethnic-racial socialization on the ethnic identity and mental health of students and the effect of ethnic identity on students’ mental health would differ (Umaña-Taylor, 2023). To date, research comparing the ethnic-cultural experiences of cultural majority and minority remains limited both in the Western and Asian literatures. To advance our knowledge, we compared the differences in the effects of students’ perception of teachers’ cultural and multicultural socialization on students’ ethnic identity and mental health in Yi and Han young people in this research.

### The study context

China has 56 ethnic groups, with the Han people being the dominant group, representing over 90% of the population (National Bureau of Statistics of China, 2021). Ethnic minorities in China are indigenous peoples who mostly reside in the remote and less wealthy parts of China’s border regions (Postiglione, 2017). China’s ethnic-minority policy advocates “pluralism within unity”; that is, cultural diversity is celebrated if national unity is upheld (Postiglione, 2017; Sude and Dervin, 2020). As such, the discourse on race and ethnicity in China focuses on harmony and integration rather than discrimination and oppression (Sude and Dervin, 2020). Since the 1950s, various autonomous regions for ethnic minorities have been established in different parts of China to provide them with independence in managing internal affairs in their own regions under the unified leadership of the Chinese government (Postiglione, 2017). However, racial inequality remains prevalent. Research has demonstrated education and health inequality between the mainstream Han people and ethnic minorities at the country level (e.g., Chen et al., 2015; Sude and Dervin, 2020).

In Liangshan, the Yi ethnic community also experienced disadvantages despite being the ethnic majority in the region, comprising 54.6% of the prefecture’s total population (People’s Government of Liangshan Yi Autonomous Prefecture, 2021). The Yi used to be a nomadic pastoral community with their own cultural practices, languages, festivals, folklore, and religion (Harrell, 2001). As China modernized, the Yi remained ethnically unmixed and retained their cultural practices due to the area’s remoteness, resulting in impoverishment (Harrell, 2001). Chronic poverty has led to a high percentage of parental loss and instability, leading to maladaptive development, such as the occurrence of post-traumatic symptoms in Yi children (e.g., Lai et al., 2022a).

Furthermore, the history of Han superiority has led to the Yi’s self-stigmatization in their own land (Harrell, 2001). Quantitative studies in young Yi people have shown weakening sense of ethnicity over time after they started school, which is heavily influenced by a Han dominated curriculum (Lai and Wong, 2019). Data from an unpublished report in 2019 also showed that Yi secondary school students tended to have higher levels of depressive symptoms, weaker ethnic identity, and poorer perceived teacher–student relationships than their Han counterparts. Follow-up qualitative research suggested that Yi students felt that they were “backward” and “inferior” compared to the Han majority in their schools (Lai et al., 2023).

On the other hand, studies suggested that the Yi people’s unique cultural roots could become their psychological asset: Strong ethnic identity has been proven to improve the psychological outcomes of Yi youths, namely, their higher levels of academic motivation and self-efficacy (Lai et al., 2019, 2022b). Qualitative findings further demonstrated the benefits of school cultural socialization and multicultural socialization on Yi adolescents’ ethnic identity, such as understanding their ethnic hardships and racial equality in class, developing an attachment with their ethnic peers via cultural participation, and pledging to contribute back to the Yi community through integrating Han and Yi practices (Lai et al., 2023). Hence, promoting supportive ethnic-racial socialization in school settings, becomes one way to enhance the ethnic identity development and mental health of young Yi people.

For Han-majority university students, strong ethnic identity was related to their positive ethnic interaction attitudes (Chen et al., 2022). Another study comparing the ethnic identity and intergroup attitudes of Han and Yi students showed that, for Han students, strong ethnic identity was positively correlated with their national identity, which, in turn, was associated with their higher levels of intergroup attitudes (Guan et al., 2022a). Alternatively, for Yi students, ethnic identity was directly associated with their positive intergroup attitudes (Guan et al., 2022a). Furthermore, multicultural socialization via teaching ethnic music to Han and Yi students was shown to be effective in promoting the ethnic identity of both groups (Guan et al., 2022b). Guan et al.’s (2022a) research provided preliminary evidence that ethnic identity carried distinct meanings to young people based on their dissimilar cultural status in China. On the other hand, supportive school ethnic-racial socialization could enhance students’ sense of ethnic identity regardless of their cultural roots (Guan et al., 2022b).

### Conceptual model, research questions, and hypotheses

Using Liangshan as an example, this study examined the effects of students’ perception of teachers’ Yi cultural socialization, Han cultural socialization, and cultural diversity attitudes on the ethnic identity and mental health of Yi and Han students. For Yi students, Han cultural socialization was a form of multicultural socialization, and Yi cultural socialization was their experiences of being socialized to their own culture. Similarly, for Han students, Yi cultural socialization was their experience of multicultural socialization, and Han cultural socialization as the practices of socializing them to their own culture.

**Figure 1:**
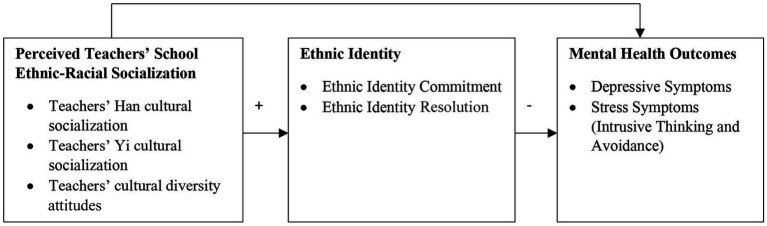
Conceptual model.

In the conceptual model (Figure 1), students’ perception of teachers’ ethnic-racial socialization was conceptualized as an endogenous variable affecting ethnic identity, and ethnic identity as a potential mediating variable linking students’ perception of teachers’ ethnic-racial socialization and those students’ mental health. We did not test the mediating effect of ethnic identity due to the cross-sectional nature of the data. To compare the experiences of Yi and Han students, the conceptual model was tested in Yi and Han students separately.

The research questions and relevant hypotheses were as follows:

Research question 1: What were the associations among students’ perception of teachers’ ethnic-racial socialization, students’ ethnic identity and mental health in Yi youths?

*Hypotheses 1.1 and 1.2*: Students’ perception of teachers’ Yi cultural socialization were positively associated with ethnic identity commitment and ethnic identity resolution.

*Hypotheses 1.3 and 1.4*: Students’ perception of teachers’ Han cultural socialization were positively associated with ethnic identity commitment and ethnic identity resolution.

*Hypotheses 1.5 and 1.6*: Students’ perception of teachers’ cultural diversity attitudes was positively associated with ethnic identity commitment and ethnic identity resolution.

*Hypotheses 1.7, 1.8 and 1.9*: Students’ perception of teachers’ Yi cultural socialization, students’ perception of teachers’ Han cultural socialization, and students’ perception of teachers’ cultural diversity attitudes were negatively associated with the mental health indicators of depressive and stress symptoms.

*Hypotheses 1.10 and 1.11*: Ethnic identity commitment and ethnic identity resolution were negatively associated with the mental health indicators of depressive and stress symptoms.

Research question 2. What were the associations among students’ perception of teachers’ ethnic-racial socialization, students’ ethnic identity and mental health in Han youths?

*Hypotheses 2.1 and 2.2*: Students’ perception of teachers’ Yi cultural socialization were positively associated with ethnic identity commitment and ethnic identity resolution.

*Hypotheses 2.3 and 2.4*: Students’ perception of teachers’ Han cultural socialization were positively associated with ethnic identity commitment and ethnic identity resolution.

*Hypotheses 2.5 and 2.6*: Students’ perception of teachers’ cultural diversity attitudes were positively associated with ethnic identity commitment and ethnic identity resolution.

*Hypotheses 2.7, 2.8, and 2.9*: Students’ perception of teachers’ Yi cultural socialization, students’ perception of teachers’ Han cultural socialization, and students’ perception of teachers’ cultural diversity attitudes were negatively associated with the mental health indicators of depressive and stress symptoms.

*Hypotheses 2.10 and 2.11*: Ethnic identity commitment and ethnic identity resolution were negatively associated with the mental health indicators of depressive and stress symptoms.

Research question 3. Did the associations among students’ perception of teachers’ ethnic-racial socialization, students’ ethnic identity and mental health differ between Yi and Han students?

*Hypothesis 3.1 and Hypothesis 3.2*: The effects of students’ perception of teachers’ Yi cultural socialization on ethnic identity commitment and ethnic identity resolution differed between Yi and Han students.

*Hypothesis 3.3 and Hypothesis 3.4*: The effects of students’ perception of teachers’ Han cultural socialization on ethnic identity commitment and ethnic identity resolution differed between Yi and Han students.

*Hypothesis 3.5 and Hypothesis 3.6*: The effects of students’ perception of teachers’ cultural diversity attitudes on ethnic identity commitment and ethnic identity resolution differed between Yi and Han students.

*Hypotheses 3.7, 3.8, and 3.9*: The effects of students’ perception of teachers’ Yi cultural socialization, Han cultural socialization, and cultural diversity attitudes on the mental health indicators of depressive and stress symptoms differed between Yi and Han students.

*Hypothesis 3.10 and Hypothesis 3.11*: The effects of ethnic identity commitment and ethnic identity resolution on the mental health indicators of depressive and stress symptoms differed between Yi and Han students.

## Materials and methods

A cross-sectional survey was used to collect information during the summer of 2021.

### Participants and sampling

Participants (*N* = 585; Yi students, *n* = 295; Han students, *n* = 290), from five secondary schools located in Liangshan, were invited to complete a paper-and-pen survey in the summer of 2021. Multistage random sampling was used to recruit the participants. A local psychological association providing school-based counseling services for schools in Liangshan assisted with the data collection process. First, the team randomly selected five schools from those served by the psychological association’s network. The association was serving a total of 12 schools at the time of the study. Second, one class of students from each lower secondary grade (i.e., grades 7–9) from each participating school was randomly selected to complete the survey. A total of 15 classes of students filled out the survey. The school management suggested the team to administer the survey by class because this arrangement made the process more feasible than randomly selecting individual students to fill out the questionnaire.

### Data collection procedures

Ethical approval was obtained from the corresponding university (HSEARS 20210823003) before the study. The team contacted the management of the selected schools and sought their consent to participate in the research. The team explained the purpose of the research as well as the potential benefits and risks of completing the survey to school management. After obtaining school management’s consent, the team traveled to these schools to meet with each class of students in person to administer the survey. Informed consent was obtained from the students and their guardians before conducting the survey. The survey required approximately 15 min to complete. As a token of appreciation, each student received a stationery set after completing the survey.

### Measurements

The scales used in this study were adapted from standardized measurements originally developed in English. Items measuring students’ perception of teachers’ ethnic-racial socialization and cultural diversity attitudes were modified to fit the context of rural China’s schools based on previous pilot interviews with Yi and Han school children. The students were asked to report their perceptions of their class head teachers’ ethnic-racial socialization in corresponding questionnaires. All scales were translated into Chinese and back-translated into English by team members familiar with the cultural context of Liangshan to check for consistency. The team also validated all scales using confirmatory factor analysis with the participants. Findings regarding the validity of the scale are reported in the Results section.

#### Depressive symptoms

The Chinese-validated version of the Patient Health Questionnaire-9 (PHQ-9; Tsai et al., 2014) was used. The scale consisted of nine items on depressive symptoms, measured on a scale ranging from 1 (*much more so than usual*) to 4 (*much less than usual*). The total score was generated by summing all of the items, with a higher total score indicating a higher risk of depression. The scale demonstrated good internal consistency (α > 0.84) and construct validity (all items with factor-loading coefficients >0.6) when tested with Chinese-speaking adolescents in Taiwan (Tsai et al., 2014).

#### Stress symptoms (avoidance and intrusive thinking)

Stress symptoms were evaluated using the Children’s Revised Impact of Event Scale-8 (CRIES; Perrin et al., 2005). The scale consisted of two subscales, avoidance and intrusive thinking, which documented the participants’ responses to stressful events. Young participants were first asked to recall a significant event in the past 2 weeks that had impacted them. They were then asked to rate their avoidance or intrusive thinking responses toward this specific negative event in the previous 2 weeks. Each subscale had four items measured on a four-point Likert scale (1 = *none*, 2 = *rarely*, 3 = *sometimes*, and 4 = *a lot*). By summing corresponding responses, total scores for avoidance and intrusive thinking were generated. A higher score indicated a higher level of stress symptoms. The scale was previously tested on Yi children and showed good reliability and validity (Lai et al., 2022a).

#### Ethnic identity

A modified Chinese version of the Multigroup Ethnic Identity Measure was used to evaluate participants’ sense of ethnic identity (Phinney, 1996). The original scale had 12 items. Phinney and Ong (2007) documented that the original 12-item scale consists of two items on ethnic participation and that these two items should be understood as ethnic behaviors rather than ethnic identity resolution. Hence, we omitted the two items pertaining to ethnic behaviors from the original scale and re-examined its psychometric properties. Thus, the modified scale focused on the components of ethnic identity commitment and resolution. The items were rated on a four-point Likert scale ranging from 1 (*strongly agree*) to 4 (*strongly disagree*). The total score was calculated by summing the responses. Higher scores indicated a stronger ethnic identity. The scale was previously tested with Yi children and adolescents and demonstrated good validity and reliability (Lai et al., 2019, 2022a,b).

#### Students’ perception of teachers’ Yi cultural socialization

The Chinese version of the Cultural Socialization Scale was used (Wang et al., 2015). This scale was originally designed to measure family and peer cultural heritage socialization based on the ethnic-racial socialization framework (Hughes et al., 2006). As no standardized scale was available to measure teachers’ cultural socialization, we adopted Wang et al.’s scale. The scale was reworded to explore teachers’ practices in engaging students in Yi cultural learning. For example, in the Cultural Socialization Scale for Yi students, one of the items was phrased as: “My teacher will teach me about Yi culture and history,” with the word “Yi” added to highlight the scale’s cultural orientation. Each subscale consisted of six items, with responses ranging from 1 (*never*) to 5 (*always*). Higher total scores indicated higher levels of cultural and mainstream socialization. The total score for each subscale was generated by summing all six items. For Yi students, this scale measured their teachers’ cultural hertiage socialization practices; for Han students, this scale measured their teachers’ practices of socializing them to the other dominant culture in Liangshan, that is, the Yi culture.

#### Students’ perception of teachers’ Han cultural socialization

The Chinese version of the Cultural Socialization Scale was used to measure teachers’ Han cultural socialization (Wang et al., 2015). The scale was reworded to pertain to the teachers’ practices in engaging students in Han cultural learning. Each subscale consisted of six items, with responses ranging from 1 (*never*) to 5 (*always*). Higher total scores indicated higher levels of cultural and mainstream socialization. The total score for each subscale was generated by summing all six items. For Han students, this scale measured their teachers’ cultural heritage socialization practices; for Yi students, this scale measured their teachers’ practices of socializing them to the other dominant culture in Liangshan, that is, the Han culture.

#### Students’ perception of teachers’ cultural diversity attitudes

The modified Chinese version of the Teachers’ Attitude on Adoption of Cultural Diversity Scale was used to measure students’ perception of teachers’ cultural diversity attitudes (Yildirim and Tezci, 2016). The original scale consisted of 16 items. As the scale was originally designed as a teacher-report scale, we modified some items to create a student-self-report version. Five items were selected from this scale to measure students’ perceptions of teachers’ attitudes toward cultural diversity. We omitted the remaining 11 items first to maintain the conciseness of the survey; more importantly, these 11 items were designed such that only the teachers themselves could answer them. The responses ranged from 1 (*strongly disagree*) to 5 (*strongly agree*). The total score was generated by adding the five items, with a higher score indicating a better attitude.

### Data analysis

A two-step structural equation model, multi-group confirmatory factor analysis (CFA), and multigroup modeling were performed using the statistical software R. First, a measurement model was built with all of the latent variables, and a CFA was performed to test the validity of the overall measurement model. To compare the differential effects of the various parameters estimated in the Yi and Han students, multigroup modeling was used.

The first step in multigroup modeling was to examine the measurement invariance across Yi and Han students in the CFA. This ensured that the subsequent findings were comparable between the two groups of students. Each scale was fitted in a multigroup CFA model for configural invariance testing, that is, an equal form of factor structure across groups, followed by models with metric invariance, that is, no significant difference in the factor loadings across groups. For model fit indices, the combination of the following cut-offs was used: close to 0.95 for CFI/TLI, 0.06 for RMSEA, and 0.05 for SRMR (Hu and Bentler, 1999; Byrne, 2001). Differences in the model fit indices were noted when assessing which invariance model fit the data better: CFI/TLI (< −0.01), RMSEA (< 0.01), and SRMR (< 0.01; Chen, 2007).

In the second step of the modeling, the covariances between the latent variables were re-specified as direct effects. The errors were correlated based on theoretical assumptions to improve the fit index of the model. Age and sex were controlled for in the final Structural Equation Model (SEM). The difference in the strength of all of the estimated parameters between Yi and Han was examined using Wald statistics.

## Results

The participants were 585 secondary school students in Liangshan aged between 13 and 15 years (*M* = 14.09), with 60.7% being female and 39.3% being male. Table 1 presents the descriptive statistics of the Yi and Han students.

**Table 1:** Descriptive statistics.

	All Students *N* = 585	Yi Students *N* = 295	Han Students *N* = 253	Missing *N*
Depression	17.21 (4.69)	17.58 (4.77)	16.83 (4.58)	6
Avoidance	10.44 (3.60)	10.07 (3.56)	10.81 (3.61)	0
Intrusive thinking	11.12 (3.51)	10.85 (3.54)	11.41 (3.47)	0
Ethnic identity—commitment	16.55 (2.60)	16.48 (2.74)	16.63 (2.47)	1
Ethnic Identity—resolution	15.26 (2.56)	15.58 (2.56)	14.93 (2.53)	1
Students’ perception of teachers’ cultural socialization of Yi	23.79 (4.56)	23.97 (4.89)	23.60 (4.21)	0
Students’ perception of teachers’ cultural socialization of Han	25.04 (4.02)	24.88 (4.06)	25.22 (3.99)	0
Students’ perception of teachers’ cultural diversity attitudes	16.48 (2.79)	16.30 (2.94)	16.55 (2.63)	0
Age	14.08 (0.77)	14.29 (0.73)	13.87 (0.76)	0
Gender	Female = 230 Male = 355	Female = 202 Male = 93	Female = 137 Male = 153	0

The covariance matrix and the means, skewness, and kurtosis values of the data are available as Supplementary materials for reproduction of the analyzes and results; the data matrix was positive definite.

### Measurement models

The overall CFA model showed satisfactory fit statistics (CFI = 0.91, TLI = 0.90, RMSEA = 0.05, SRMR = 0.05). Table 2 lists the factor loadings of the model. The average loading was over 0.70 for the Yi and Han model, which was satisfactory (Cabrera-Nguyen, 2010).

**Table 2:** *z* values, standardized factor loadings, 95% confidence intervals, and standard errors of the overall multigroup CFA model of Yi and Han samples.

Item	Yi (*n* = 295)					Han (*n* = 290)				
	*z*	Estimates	95% CI		*SE*	*z*	Estimates	95% CI		*SE*
			LL	UL				LL	UL	
HS1 Encourage me to respect Han cultural values and beliefs	36.252	0.824	0.779	0.868	0.023	52.484	0.862	0.829	0.894	0.016
HS2 Teach me about Han cultural values and beliefs	35.724	0.821	0.776	0.866	0.023	95.285	0.928	0.908	0.947	0.010
HS3 Talk to me about the importance about understanding Han cultural background	35.621	0.820	0.775	0.865	0.023	97.865	0.930	0.911	0.948	0.010
HS4 Teach to me about the history of Han peoples	22.909	0.721	0.659	0.783	0.031	96.720	0.929	0.910	0.948	0.010
HS5 Participate in activities that are specific to Han cultures	24.254	0.737	0.677	0.797	0.030	62.351	0.884	0.856	0.912	0.014
HS6 Attend concerts, festivals, or other events related to Han cultures	23.684	0.731	0.671	0.792	0.031	41.174	0.826	0.786	0.865	0.020
YS1 Encourage me to respect Yi cultural values and beliefs	40.299	0.833	0.792	0.873	0.021	42.906	0.832	0.794	0.870	0.019
YS2 Teach me about Yi cultural values and beliefs	40.597	0.834	0.794	0.874	0.021	98.167	0.932	0.913	0.950	0.009
YS3 Talk to me about the importance about understanding Yi cultural background	66.285	0.910	0.883	0.937	0.014	92.631	0.927	0.907	0.946	0.010
YS4 Teach to me about the history of Yi peoples	41.918	0.840	0.801	0.879	0.020	107.690	0.940	0.923	0.957	0.009
YS5 Participate in activities that are specific to Yi cultures	24.083	0.726	0.667	0.785	0.030	27.163	0.745	0.691	0.799	0.027
YS6 Attend concerts, festivals, or other events related to Yi cultures	18.923	0.665	0.596	0.734	0.035	27.765	0.750	0.697	0.803	0.027
CD1 Respect and give importance to cultural diversity	20.658	0.715	0.648	0.783	0.035	20.777	0.710	0.643	0.777	0.034
CD2 Care about students’ description of their cultural values	22.801	0.743	0.679	0.807	0.033	31.280	0.816	0.764	0.867	0.026
CD3 In classroom, teachers’ activities care about students’ cultural diversity	23.879	0.756	0.694	0.818	0.032	26.950	0.779	0.722	0.835	0.029
CD4 Teachers encourage respect and acceptance of different cultures during in-class debates	23.836	0.755	0.693	0.817	0.032	28.443	0.792	0.737	0.847	0.028
RES1 I have spent time trying to find out more about my ethnic group, such as its history, traditions, and customs	14.462	0.611	0.528	0.694	0.042	15.799	0.638	0.559	0.717	0.040
RES2 I have a clear sense of my ethnic background and what it means for me	8.184	0.432	0.328	0.535	0.053	10.746	0.517	0.423	0.612	0.048
RES3 I think a lot about how my life will be affected by my ethnic group membership	17.117	0.662	0.586	0.738	0.039	24.209	0.760	0.698	0.821	0.031
RES4 I understand pretty well what my ethnic group membership means to me	22.731	0.744	0.680	0.808	0.033	19.768	0.703	0.634	0.773	0.036
RES5 In order to learn more about my ethnic background, I have often talked to other people about my ethnic group	23.063	0.748	0.685	0.812	0.032	25.891	0.778	0.719	0.836	0.030
COM1 I am happy that I am a member of the group I belong to	20.914	0.705	0.639	0.771	0.034	35.893	0.828	0.783	0.873	0.023
COM2 I have a strong sense of belonging to my own ethnic group	31.753	0.808	0.758	0.857	0.025	34.129	0.818	0.771	0.865	0.024
COM3 I have a lot of pride in my ethnic group	25.315	0.754	0.696	0.813	0.030	26.964	0.765	0.710	0.821	0.028
COM4 I feel a strong attachment to my own ethnic group	24.451	0.746	0.686	0.805	0.030	19.490	0.683	0.614	0.752	0.035
COM5 I feel good about my cultural or ethnic background	18.345	0.669	0.598	0.741	0.036	21.709	0.712	0.647	0.776	0.033
DEP1 Little interest or pleasure in doing things	14.020	0.614	0.528	0.700	0.044	14.932	0.631	0.548	0.714	0.042
DEP2 Feeling down, depressed, or hopeless	19.906	0.725	0.653	0.796	0.036	17.023	0.672	0.595	0.749	0.039
DEP3 Trouble falling or staying asleep, or sleeping too much	11.450	0.548	0.454	0.642	0.048	13.161	0.591	0.503	0.679	0.045
DEP4 Feeling tired or having little energy	15.144	0.639	0.556	0.721	0.042	15.246	0.638	0.556	0.720	0.042
DEP5 Poor appetite or overeating	13.561	0.603	0.516	0.690	0.044	12.275	0.569	0.478	0.660	0.046
DEP6 Feeling bad about self—or that you are a failure or have let yourself or your family down	2.956	0.186	0.063	0.309	0.063	4.552	0.274	0.156	0.393	0.060
DEP7 Trouble concentrating on things, such as reading the newspaper or watching television	7.266	0.403	0.295	0.512	0.056	6.485	0.369	0.257	0.480	0.057
DEP8 Moving or speaking so slowly that other could have noticed? Or the opposite—being so fidgety or restless that you have been moving around a lot more than usual	9.643	0.493	0.392	0.593	0.051	12.476	0.574	0.484	0.664	0.046
DEP9 Thoughts that you would be better off dead, or of hurting yourself in some way	10.968	0.534	0.439	0.630	0.049	14.752	0.627	0.544	0.711	0.043
IT1 Do you think about it even when you do not mean to?	37.973	0.841	0.797	0.884	0.022	30.942	0.798	0.748	0.849	0.026
IT2 Do you have waves of strong feelings about it?	28.480	0.778	0.724	0.831	0.027	37.397	0.839	0.795	0.883	0.022
IT3 Do other things keep making you think about it?	43.350	0.868	0.829	0.907	0.020	39.661	0.852	0.810	0.894	0.021
IT4 Do pictures about it pop into your mind?	21.613	0.710	0.646	0.774	0.033	25.467	0.753	0.695	0.811	0.030
AVO1 Do you try not to talk about it?	18.838	0.695	0.623	0.768	0.037	22.467	0.739	0.675	0.804	0.033
AVO2 Do you try to remove it from your memory?	20.292	0.717	0.648	0.787	0.035	27.694	0.796	0.740	0.853	0.029
AVO3 Do you stay away from reminders of it (e.g., places or situations)?	22.340	0.745	0.680	0.811	0.033	21.837	0.732	0.666	0.797	0.034
AVO4 Do you try not to think about it?	22.779	0.751	0.686	0.815	0.033	24.765	0.766	0.705	0.827	0.031

HS, teachers’ Han socialization; YS, teachers’ Yi socialization; CD, teachers’ cultural diversity attitudes; RES, ethnic identity resolution; COM, ethnic identity commitment; DEP, depression; IT, intrusive thinking; AVO, avoidance; CI, confidence intervals; LL, lower limit; UL, upper limit. All loadings are significant at *p* < 0.001 except DEP6 at *p* = 0.003.

### Measurement invariance

Table 3 showed the model fit indices of the multigroup CFA models. The data fit indices were acceptable for all configural invariances, suggesting that the measurements had an equal structure for the Yi and Han students. In general, the data achieved metric invariance across both groups of students with acceptable fit indices. Although the changes in the fit indices of the SRMR for teachers’ Han cultural socialization, teachers’ cultural diversity attitudes, ethnic identity exploration, and avoidance marginally exceeded the cutoff for TLI and SRMR (i.e., larger than 0.01), the changes in CFI and RMSEA did not. Ethnic identity commitment and intrusive thinking were not metrically invariant across Yi and Han students, suggesting that the factor loadings of the items differed between the two groups. Considering that the two variables reached configural invariance, it was acceptable for them to have dissimilar factor loadings.

**Table 3:** Model fit indices and their differences between multigroup CFA and measurement invariance models of all variables.

Variable	CFI	TLI	Configural invariance RMSEA	SRMR	CFI	Metric TLI	Invariance RMSEA	SRMR	Scalar invariance			
									CFI	TLI	RMSEA	SRMR
Teachers’ Han socialization	0.950	0.917	0.171	0.029	0.948	0.932	0.155	0.048	0.948	0.944	0.141	0.049
Δ	–	–	–	–	−0.002	0.015	−0.016	0.019	0.000	0.012	−0.014	0.001
Teachers’ Yi socialization	0.941	0.901	0.184	0.041	0.941	0.923	0.163	0.046	0.934	0.930	0.155	0.052
Δ	–	–	–	–	0.000	0.022	−0.021	0.005	−0.007	0.007	−0.008	0.006
Teachers’ cultural diversity attitudes	0.983	0.948	0.118	0.019	0.983	0.970	0.089	0.030	0.979	0.975	0.082	0.036
Δ	–	–	–	–	0.000	0.022	−0.029	0.011	−0.004	0.005	−0.007	0.006
Ethnic identity commitment	0.943	0.887	0.128	0.036	0.931	0.901	0.119	0.056	0.914	0.905	0.117	0.064
Δ	–	–	–	–	−0.012	0.014	−0.009	0.020	−0.017	0.004	−0.002	0.008
Ethnic identity resolution	0.965	0.929	0.126	0.030	0.961	0.945	0.111	0.045	0.953	0.948	0.108	0.051
Δ	–	–	–	–	−0.004	0.016	−0.015	0.015	−0.008	0.003	−0.003	0.006
Depression	0.960	0.946	0.052	0.037	0.961	0.955	0.048	0.044	0.942	0.940	0.055	0.051
Δ	–	–	–	–	0.001	0.009	−0.004	0.007	−0.019	−0.015	0.007	0.007
Intrusive thinking	0.999	0.998	0.027	0.009	0.996	0.992	0.052	0.037	0.996	0.996	0.039	0.038
Δ	–	–	–	–	−0.003	−0.006	0.025	0.028	0.000	0.004	−0.013	0.001
Avoidance	0.990	0.971	0.084	0.017	0.991	0.985	0.061	0.027	0.992	0.990	0.050	0.030
Δ	–	–	–	–	0.001	0.014	−0.023	0.010	0.001	0.005	−0.011	0.003

Δ, differences in fits against the previous invariance model. Yi *n* = 295; Han *n* = 290.

### Structural equation models

**Figure 2:**
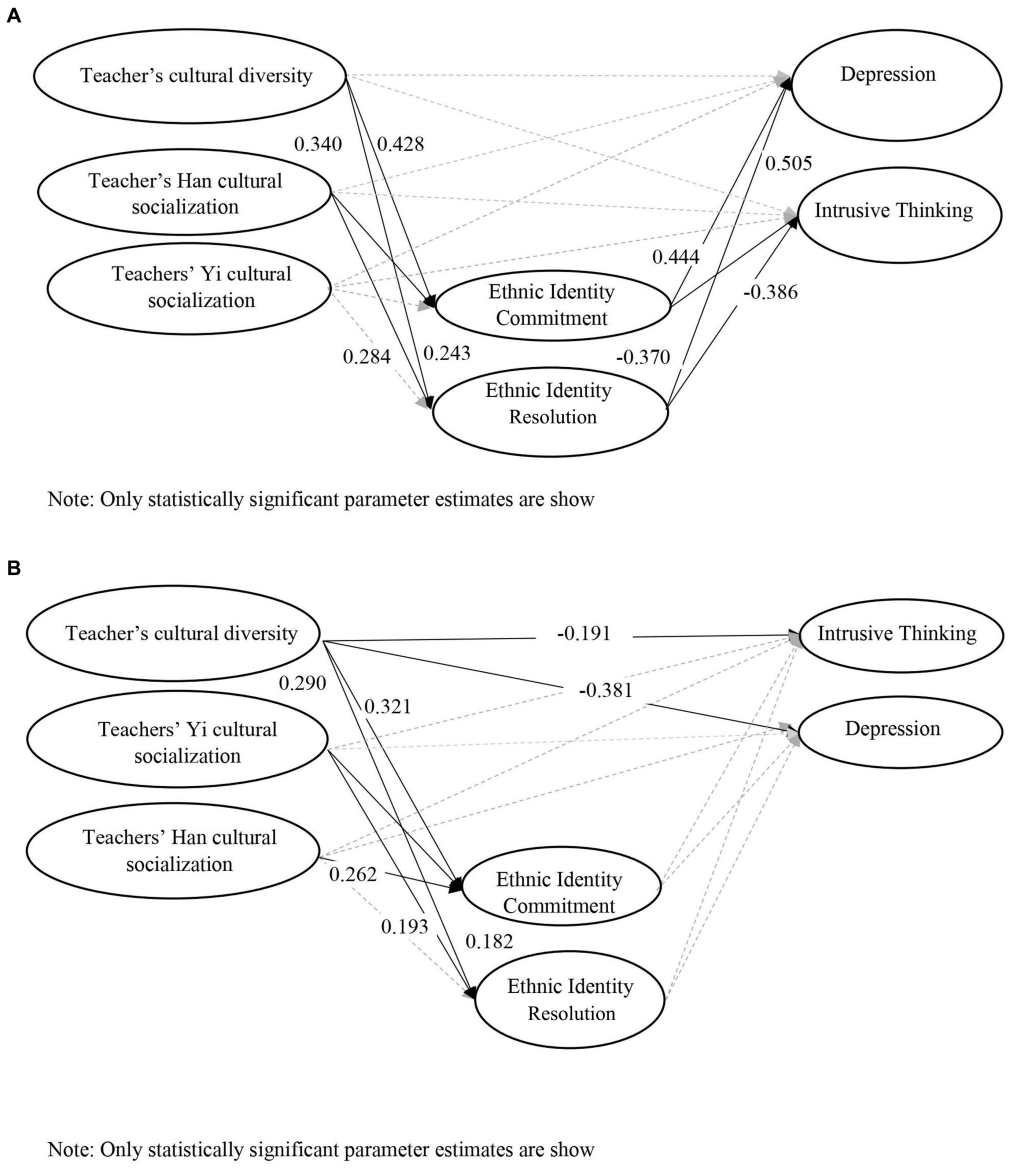
**(A)** The pathway from students’ perception of teachers’ ethnic-racial socialization to ethnic identity and mental health in Yi students. **(B)** The pathway from students’ perception of teachers’ ethnic-racial socialization to ethnic identity and mental health in Han students.

Table 4 shows the standardized estimates of the direct effects in Yi and Han students and the differences in the parameter effects between the two samples based on the Wald statistics. Figures 2A,B showed the statistically significant pathways in the Yi and Han models, respectively. The following first summarized the findings in Yi students, followed by Han students, and finally, a comparison on the different effects of the pathways among the variables between the two ethnic groups.

**Table 4:** *z* values, standardized estimates, 95% confidence intervals, standard errors, and *p* values of direct paths in the multigroup SEM model and their differences between Yi and Han samples after controlling for gender and age.

Direct path	Yi (*n* = 295)					Han (*n* = 290)					Difference between Yi and Han				
	*z*	Estimates	95% CI		*SE*	*z*	Estimates	95% CI		*SE*	*z*	Estimates	95% CI		*SE*
			LL	UL				LL	UL				LL	UL	
HS → RES	3.057	0.284*	0.102	0.466	0.093	1.732	0.169	−0.022	0.360	0.097	0.856	0.115	−0.149	0.379	0.135
YS → RES	0.574	0.048	−0.116	0.212	0.084	2.001	0.193*	0.004	0.381	0.096	−1.134	−0.145	−0.394	0.105	0.127
CD → RES	3.702	0.340*	0.160	0.520	0.092	3.791	0.290*	0.140	0.440	0.076	0.420	0.050	−0.184	0.284	0.120
HS → COM	2.762	0.243*	0.071	0.416	0.088	3.061	0.262*	0.094	0.429	0.085	−0.149	−0.018	−0.259	0.222	0.123
YS → COM	0.257	0.020	−0.134	0.175	0.079	2.144	0.182*	0.016	0.349	0.085	−1.396	−0.162	−0.389	0.065	0.116
CD → COM	5.010	0.428*	0.261	0.596	0.085	4.785	0.321*	0.190	0.453	0.067	0.984	0.107	−0.106	0.320	0.109
HS → DEP	−0.519	−0.056	−0.268	0.156	0.108	−0.806	−0.088	−0.303	0.126	0.110	0.210	0.032	−0.269	0.334	0.154
YS → DEP	−0.790	−0.074	−0.256	0.109	0.093	1.292	0.138	−0.071	0.348	0.107	−1.493	−0.212	−0.489	0.066	0.142
CD → DEP	−1.037	−0.119	−0.345	0.106	0.115	−4.260	−0.381*	−0.556	−0.206	0.089	1.791	0.261	−0.025	0.547	0.146
RES → DEP	−2.110	−0.370*	−0.714	−0.026	0.175	0.492	0.064	−0.190	0.318	0.130	−1.990	−0.434	−0.862	−0.007	0.218
COM → DEP	2.525	0.444*	0.099	0.788	0.176	−0.480	−0.068	−0.348	0.211	0.143	2.263	0.512*	0.069	0.956	0.226
HS → IT	0.848	0.090	−0.118	0.297	0.106	0.268	0.030	−0.189	0.249	0.112	0.390	0.060	−0.242	0.361	0.154
YS → IT	0.486	0.044	−0.135	0.223	0.091	0.643	0.070	−0.143	0.283	0.109	−0.180	−0.026	−0.304	0.253	0.142
CD → IT	−1.306	−0.148	−0.369	0.074	0.113	−2.050	−0.191*	−0.374	−0.008	0.093	0.297	0.043	−0.244	0.331	0.147
RES → IT	−2.237	−0.386*	−0.724	−0.048	0.173	−0.164	−0.022	−0.280	0.237	0.132	−1.678	−0.364	−0.790	0.061	0.217
COM → IT	2.930	0.505*	0.167	0.842	0.172	0.540	0.078	−0.206	0.363	0.145	1.893	0.426	−0.015	0.868	0.225
HS → AVO	1.291	0.143	−0.074	0.361	0.111	1.343	0.151	−0.070	0.372	0.113	−0.049	−0.008	−0.318	0.302	0.158
YS → AVO	−0.328	−0.032	−0.220	0.157	0.096	−0.050	−0.005	−0.222	0.211	0.110	−0.178	−0.026	−0.313	0.261	0.146
CD → AVO	−0.140	−0.017	−0.250	0.217	0.119	−0.951	−0.090	−0.277	0.096	0.095	0.484	0.074	−0.225	0.372	0.152
RES → AVO	−1.326	−0.239	−0.592	0.114	0.180	1.182	0.158	−0.104	0.419	0.133	−1.769	−0.396	−0.836	0.043	0.224
COM → AVO	1.603	0.289	−0.064	0.642	0.180	−0.400	−0.059	−0.347	0.229	0.147	1.495	0.348	−0.108	0.804	0.233
Gender→RES	−1.697	−0.094	−0.202	0.015	0.055	−0.512	−0.028	−0.138	0.081	0.056	−0.833	−0.065	−0.219	0.088	0.078
Gender→COM	−1.380	−0.072	−0.174	0.030	0.052	1.202	0.059	−0.037	0.155	0.049	−1.828	−0.131	−0.271	0.009	0.072
Gender→DEP	5.269	0.311*	0.195	0.427	0.059	3.032	0.184*	0.065	0.303	0.061	1.500	0.127	−0.039	0.293	0.085
Gender→IT	3.721	0.222*	0.105	0.339	0.060	0.070	0.004	−0.119	0.128	0.063	2.502	0.217	0.047	0.388	0.087
Gender→AVO	1.876	0.120	−0.005	0.246	0.064	0.911	0.058	−0.067	0.183	0.064	0.686	0.062	−0.115	0.239	0.091
Age→RES	0.785	0.044	−0.065	0.152	0.056	1.277	0.071	−0.038	0.180	0.056	−0.348	−0.027	−0.181	0.127	0.079
Age→COM	0.139	0.007	−0.095	0.110	0.052	2.225	0.109*	0.013	0.204	0.049	−1.416	−0.101	−0.242	0.039	0.072
Age→DEP	−0.007	0.000	−0.123	0.122	0.062	0.867	0.053	−0.067	0.174	0.062	−0.614	−0.054	−0.226	0.118	0.088
Age→IT	−1.129	−0.069	−0.189	0.051	0.061	0.575	0.036	−0.087	0.159	0.063	−1.199	−0.105	−0.277	0.067	0.088
Age→AVO	−0.716	−0.046	−0.172	0.080	0.064	1.942	0.123	−0.001	0.246	0.063	−1.871	−0.169	−0.346	0.008	0.090

HS, teachers’ Han socialization; YS, teachers’ Yi socialization; CD, teachers’ cultural diversity attitudes; RES, ethnic identity resolution; COM, ethnic identity commitment; DEP, depression; IT, intrusive thinking; AVO, avoidance; CI, confidence intervals; LL, lower limit; UL, upper limit. Females were coded as 1, and males as 0.

#### Students’ perception of teachers’ ethnic-racial socialization, and ethnic identity and mental health in Yi students

Regarding the mental health of Yi students, their perception of teacher cultural diversity attitudes and Han and Yi cultural socialization did not show any direct effects on Yi students’ depression or stress symptoms. On the other hand, their ethnic identity commitment was associated with higher levels of depression and stress symptoms of intrusive thinking, whereas ethnic identity resolution led to lower levels of depression and intrusive thinking. No effect on avoidant thinking was observed.

For the ethnic identity outcomes of Yi students, students’ perception of teachers’ multicultural socialization practices, that is, Han cultural socialization and cultural diversity attitudes, had positive effects on their ethnic identity resolution and commitment. Alternatively, their perception of teachers’ socialization regarding their own culture, that is, Yi cultural socialization, did not affect their ethnic identity commitment and resolution.

Synthesizing these findings, ethnic identity served as a linking variable between students’ perceptions of their teachers’ multicultural socialization practices and their mental health outcomes of depression and intrusive thinking. Students’ perception of teachers’ multicultural socialization increased their ethnic identity resolution, which then decreased depressive and intrusive thinking symptoms. On the other hand, while teachers’ multicultural socialization also enhanced ethnic identity commitment, it was associated with higher levels of depressive symptoms and intrusive thinking.

#### Students’ perception of teachers’ ethnic-racial socialization, ethnic identity, and mental health in Han students

Regarding the mental health of Han students, their perception of teachers’ cultural diversity attitudes was associated with lower levels of depression and intrusive thinking. However, neither students’ perception of teachers’ Yi and Han cultural socialization nor the component of ethnic identity correlated with any mental health outcomes. No effect on avoidance thinking was observed.

For the ethnic identity outcomes of Han students, students’ perception of teachers’ multicultural socialization, that is, Yi cultural socialization and cultural diversity attitudes, had positive effects on their ethnic identity resolution and commitment. Teachers’ socialization regarding the students’ own culture, that is, Han cultural socialization, had a positive effect only on their ethnic identity commitment.

As ethnic identity did not have an effect on the mental health of Han students, it did not serve as a linking variable between students’ perception of teachers’ ethnic-racial socialization and mental health outcomes in Han students.

#### Differences between Yi and Han students

The findings of the test of differences in specific pathways showed that the effects of ethnic identity commitment and resolution on depression were significantly different between Yi students and Han students. While no statistically significant differences were identified between Yi and Han students in the following pathways, these effects showed practical significance, as supported by existing theories. These pathways were as follows: (i) from ethnic identity commitment and resolution to intrusive thinking in Yi students only; (ii) from teachers’ cultural diversity attitudes to depression and intrusive thinking in Han students only; (iii) from teachers’ Han cultural socialization to ethnic identity commitment and resolution in Yi students only; and (iv) from teachers’ Yi cultural socialization to ethnic identity commitment and resolution in Han students only. Due to the different meaning of Han and Yi cultural socialization to Han and Yi students, pathways (iii) and (iv) can be interpreted as the positive ethnic identity effect of teachers’ socializing their students to the other dominating culture in Liangshan, whereas teachers socializing them to their own cultures did not show such effects. The statistically non-significant findings in these pathways might be explained by the high sensitivity of Wald test, causing it to fail to detect differences between the Yi versus Han participants.

## Discussion

This study examines the effects of students’ perception of teachers’ ethnic-racial socialization on their their ethnic identity and mental health. Comparing our findings of Yi and Han students, students’ perception of teachers’ ethnic-racial socialization practices have dissimilar effects on their ethnic identity and their mental health outcomes of depression and intrusive thinking. Interestingly, the stress symptom of avoidance is not related to either students’ perception of teachers’ ethnic-racial socialization or their ethnic identity in the Yi and Han group. Further research is required to understand the cross-cultural meaning of avoidance in young people in the context of China.

For the Yi students, their ethnic identity serves as a link between their perception of teachers’ multicultural socialization practices and their mental health. The students’ perception of their teachers’ multicultural socialization practice has a positive effect on their ethnic identity, and their ethnic identity is then related to their mental health outcomes. This set of findings underscores the role of ethnic identity in bridging Yi students’ external ethnic-racial socialization experiences and their internal-psyche. While ethnic identity resolution is associated with lower levels of depressive symptoms and intrusive thinking, ethnic identity commitment is related to higher levels of depression and intrusive thinking. We will discuss this interesting findings with reference to the ethnic identity theory in the latter part of this section.

For the Han students, ethnic identity is not a linking variable. Students’ perception of their teachers’ multicultural socialization practices directly improves Han students’ mental health by decreasing their depressive and intrusive thinking symptoms. It also directly affects their sense of ethnic identity. However, ethnic identity does not have an effect on their mental health. Consistent with existing literature on cultural majorities (Ison et al., 2023; Umaña-Taylor, 2023), the psychological impact of ethnic identity is lower in Han than Yi students.

In the following, we discuss the three key findings that compare the differences between the Yi versus Han students.

### Key finding 1: students’ perception of teachers’ multicultural socialization practices positively affect the ethnic identity of both Yi and Han youths, but own cultural heritage socialization does not have the same effect

Students’ perception of teachers’ multicultural socialization practices, show positive effects on ethnic identity commitment and resolution in both Yi and Han students. On the other hand, the students’ perception of teachers’ own cultural socialization, that is, the teachers’ practice of socializing the students to their own culture, does not have any effect on Yi students’ ethnic identity commitment and resolution. Yet, it shows a positive effect on Han students’ ethnic identity commitment but not their ethnic identity resolution.

For the Yi students, higher levels of their perception of teachers’ cultural diversity attitudes and Han cultural socialization are associated with their stronger ethnic identity. Teachers’ cultural diversity attitudes may have made the Yi students feel respected and appreciated as an ethnic group, thus enhancing their sense of ethnic identity. The positive effect of Han cultural socialization on Yi students’ ethnic identity can be explained by the fact that Yi students were being exposed to a different culture that inspired them to reflect on their own (Saleem and Byrd, 2021; Lai et al., 2023). An earlier study showed that Yi students who showed a better understanding of Han culture tended to be motivated to contribute to the Yi community with deeper insights into the strengths of both the Yi and Han ethnicity (Lai et al., 2023). Furthermore, in Liangshan, where Yi students are also a cultural majority, teachers might have taught Yi students to respect and appreciate Han culture, without forcing Yi youths to adopt Han practices. Our findings, which show that socialization regarding one’s own culture (i.e., Yi cultural socialization) does not influence Yi students’ ethnic identity, can be explained by the inferior status that Yi students have in Han-driven school settings (Lai et al., 2023). Yi students may find little value in learning about or practicing Yi cultural traditions in school settings and this, thus, does not contribute to their positive self-development. Future studies shall look into the intricate ethnic minority versus majority dynamic in the other autonomous regions of China to further unpack how the local socio-cultural context affects the young people ethnic identity development.

For Han students, higher levels of their perception of teachers’ cultural diversity attitudes and Yi cultural socialization were associated with their stronger ethnic identity. This contradicts existing literature in the West, which suggests that cultural diversity learning may challenge the cultural majority’s beliefs about their status in society by informing them of their role as cultural oppressors throughout history, leading to a stage of self-doubt (Hardiman and Keehn, 2012). As mentioned, racism and racial discrimination are not the dominant discourses surrounding ethnicity in China; rather, the country focuses on cultural integration and harmony (Postiglione, 2017). Multicultural socialization motivates Han students to see themselves as promoters of racial equality through downward social. On the other hand, cultural socialization to their own Han culture only affects their ethnic identity commitment. The results further suggest that the teachers’ cultural socialization only contributes to their ethnic pride and belonging without supporting the students to understand their privileged status, and possibly, their role as the cultural oppressor throughout China’s ethnic-racial history.

Further studies are needed to enrich and deepen our knowledge regarding how we can strengthen the ethnic identity of students, especially for the resolution component. Two proposed hypotheses for follow-up research are: Teachers’ multicultural socialization improves the ethnic identity of cultural-majority and -minority students. Teachers’ socialization to own culture will have different effects on the ethnic identity development of students in the cultural-majority versus -minority group. Additional qualitative research will also facilitate us in understanding the contextual reasons explaining the differences.

### Key finding 2: students’ perception of teachers’ multicultural socialization practices have different mental health effects on Yi versus Han students

Students’ perception of teachers’ cultural diversity attitudes positively affects the mental health of Han students but not that of Yi students. For Han students, teachers who embrace cultural diversity may tend to be more empathetic and accepting of their students, thus contributing to students’ improved well-being (Byrd and Chavous, 2011; Brown and Chu, 2012; Saleem and Byrd, 2021). However, for the Yi students, the effect of teachers’ attitudes toward cultural diversity may be more complicated. Previous studies have suggested that Yi students feel discriminated against in the Han-driven school settings of Liangshan (e.g., Harrell, 2001; Lai et al., 2023), which might jeopardize their mental health. Although teachers’ attitudes toward cultural diversity can contribute to their stronger ethnic identity commitment and resolution (Saleem and Byrd, 2021), Yi students may simultaneously encounter cultural assimilation within their schools or peer discrimination. Such negative experiences may damage the ability of Yi students to trust their schools, which offsets the potential positive effect of teachers’ cultural diversity attitudes on their mental health.

In contrast, having teachers that socialized students with the other dominating cultures did not affect the mental health of either group. Learning about other people cultures can stimulate students to reflect on their ethnic status in relation to the multicultural community and foster mutual respect and cultural competence via social comparison (Saleem and Byrd, 2021). However, it may not be associated with the lower occurrence of mental health problems, because the socialization process does not tackle students’ emotional health directly. Hence, in terms of mental health promotion, teachers’ cultural diversity attitudes may play a more important role than teachers’ socialization of students to different cultures.

The differential effects of teachers’ cultural diversity attitudes on mental health in Han and Yi students highlight the need to understand the interaction effects of multiple levels of school based ethnic-racial socialization experiences on adolescents’ well-being (Umaña-Taylor, 2023). Teachers, classmates, and the school climate all contribute to young people’s self-development and well-being (Saleem and Byrd, 2021). To promote positive well-being in students of mutlicultural origins, school management needs to align its multicultural policy and create a holistic and culturally embracing environment through the collaborative efforts of teachers, students, and parents (Umaña-Taylor, 2023). Based on the findings, the following hypotheses are proposed for future research: Teachers’ cultural diversity attitudes is positively associated with the positive mental health of cultural-majority students. However, in students of ethnic-minority status, the mental health effect will depend on the social context that these students are embedded in. Qualitative research is also needed to explore how teachers’ cultural diversity attitudes affects students’ ethnic identity and psychological health.

### Key finding 3: ethnic identity affects the mental health of Yi indigenous students only

Ethnic identity affects the mental health of Yi students but not that of Han students. The protective effects of ethnic identity resolution against depression and intrusive thinking in Yi students echo the existing literature that highlights ethnic identity as a psychological resource (e.g., Rivas-Drake et al., 2014; Lai et al., 2019, 2022b; Umaña-Taylor, 2023). Those who embrace their ethnic group membership, understand their experiences of discrimination, and are willing to confront these negative encounters should show better mental health, such as fewer depressive and stress symptoms (Phinney and Ong, 2007; Umana-Taylor et al., 2014). Interestingly, our Yi participants who scored high on ethnic identity commitment showed higher levels of depression and intrusive thinking. In rural China, ethnic minorities continue to experience racial disadvantages in the Han-driven socioeconomic system (e.g., Harrell, 2001; Lai et al., 2023). Those who feel proud of their culture without acknowledging their cultural hardships can experience psychological dissonance because their expectations do not match with their racial encounters in mainstream society (Tajfel and Turner, 1986; Phinney and Ong, 2007). This incongruent feeling may lead to mental health problems (Syed et al., 2013). The current finding highlights the potential harm caused by ethnic identity commitment without resolution. To resolve this agitation, ethnic-minority youth must embrace the vulnerability of their ethnic groups and genuinely understand the meaning and history of racial discrimination and oppression in their communities. With a strong sense of ethnic identity commitment and resolution, ethnicity can become a unique psychological asset for the positive development of young ethnic minorities.

However, ethnic identity does not have the same mental health effects on ethnic-majority Han students. Han students are an ethnically privileged group in China, with an education system built to accommodate their values and traditions (Sude and Dervin, 2020). As such, ethnic identity is less salient to Han students, possibly because they take their cultural status for granted (Moffitt and Rogers, 2022; Ison et al., 2023). In fact, ethnic identity can become important to the cultural majority if they are socialized to understand their ethnic privileges and responsibilities or have experienced racial inequality (Woo et al., 2019; Ison et al., 2023). For example, White students in the U.S. who experienced racial discrimination showed higher levels of ethnic identity (Woo et al., 2019). Another study also showed that White students who showed a better understanding of their ethnicity tend to have supportive familial cultural socialization experiences (Ison et al., 2023).

The current findings are consistent with Western literature discussing the meaning of ethnic identity in cultural minorities versus the majority (e.g., Woo et al., 2019; Ison et al., 2023; Umaña-Taylor, 2023). The different effects of ethnic identity on the mental health of Yi and Han students underscore the need for us to understand ethnic identity within the sociocultural context that young people are embedded in. Even within the same rural school settings in Liangshan, Yi and Han students had dissimilar cultural experiences. However, the differences are not based on their ethnic labels; rather, it is associated with their distinct school cultural encounters due to their ethnic status in the country. Those who belong to the “inferior” group are inclined to mobilize their ethnicity as a protective asset, with a higher ability to attach personal meaning to their ethnic group membership. Alternatively, the culturally privileged group does not need to use their ethnicity to protect themselves because their cultural status is taken for granted in the general society. Based on the findings, the following hypotheses are proposed for future research, which applies to both ethnic minority and cultural majority groups: Ethnic identity resolution will result in positive mental health in students who have resolved their ethnic uncertainties and found purpose in their ethnic group membership. Ethnic identity commitment without resolution can result in higher occurrence of mental health problems in those who are treated as a racially inferior group in their society.

### Limitations

The findings of this study should be interpreted in light of the following limitations. First, it only included Yi and Han students from rural secondary schools in Liangshan, thus limiting the generalizability of the findings to other ethnic-minority groups in other regions of China. Nevertheless, ethnic-minority youth in rural China are a hard-to-reach population, and the information we obtain in this research can serve as pilot evidence for us to understand the intricate relationships between ethnic-racial socialization, ethnic identity, and mental health in non-Western settings. Future research should generalize these findings to other parts of China.

Second, this research only captured the students’ perception of their teachers’ ethnic-racial socialization practices, without using any teacher-reported scale. The research team discussed with the school management about the possibility of distributing relevant scales to the teachers to obtain their responses. However, school management was a little reluctant due to the teachers’ busy schedules. After meeting with the school management, the research team decided to employ the student-reported survey only.

Third, while an earlier study showed that Yi students in Liangshan experienced discrimination (e.g., Lai et al., 2023), this study did not measure the extent of perceived discrimination in students. School management was wary of the sensitivity of including a questionnaire about ethnic-racial discrimination in the survey; thus, the team decided not to include the relevant measures. Also considering the length of the survey, the research team decided to omit these items.

Fourth, as ethnic identity and ethnic-racial socialization theories are built on the accounts of racial and ethnic minorities (Moffitt and Rogers, 2022), the use of relevant measures for the Han cultural majority may not be completely relevant. However, considering that there are no existing measures to evaluate the ethnic identity of the cultural majority and that a pilot interview had been conducted to confirm the validity of the ethnic identity measures for Han students, the use of the standardized measure of ethnic identity in the current research seemed the most appropriate.

Finally, the mediation effect of students’ ethnic identity between students’ perception of their teachers’ multicultural socialization practices and students’ mental health outcomes could not be tested using the current cross-sectional information. A longitudinal study is needed to demonstrate the temporality of the mediation effect and ascertain whether ethnic identity explains the effect of teachers’ multicultural socialization practices on students’ mental health. Nevertheless, the current findings offer preliminary information that identifies ethnic identity as the linking variable bridging ethnic-racial socialization and mental health in ethnic-minority youths for further longitudinal analysis.

### Implications

The findings of this study have research, practical, and policy implications. First, the findings highlight the potential role of ethnic identity as the central organizing principle bridging external ethnic-racial socialization experiences and mental health in ethnic-minority young people. The current results provide a solid foundation for the conceptualization of a longitudinal study supporting the explanatory role of ethnic identity in the ethnic-racial socialization experiences and mental health of ethnic-minority youths in non-Western settings. Such findings will further support the notion that ethnicity is a cultural and psychological capital of multicultural communities, thus changing current discourse that ethnic minority as a vulnerable group. Second, to a certain extent, these findings support the universality of the notions of ethnic-racial socialization and ethnic identity (Umaña-Taylor, 2023). For instance, our results showed that ethnic identity commitment led to poorer mental health in ethnic minority youths. This findings are consistent with existing notion put forth by ethnic identity development theory, namely the potential harmful effect of ethnic identity commitment in culturally oppressed groups (Tajfel and Turner, 1986; Phinney and Ong, 2007). One the other hand, our results also advocate the psychological benefits associated with ethnic identity resolution in ethnic minorities (Umana-Taylor et al., 2014). Last, the statistically non-significant effect of ethnic identity on mental health in Han majority students also support the assumption of ignorance toward ethnic identity in the cultural majority (Umaña-Taylor, 2023).

Similarly, results showing the effect of teachers’ multicultural socialization practices on ethnic identity also aligns with the theoretical logic put forth by the school ethnic-racial socialization framework (Saleem and Byrd, 2021). Nevertheless, sociocultural variations persist. For example, in China’s Han-driven system, where ethnic minorities experience self-stigmatization and marginalization (Harrell, 2001; Lai et al., 2023), own cultural socialization contributes little to the development of ethnic identity when the socio-cultural context seldom emphasizes racial discrimination and rights. In this case, our findings differs from those in Western literatures which suggest the powerful effect of own cultural socialization on ethnic identity among ethnic minority youths (e.g., Byrd, 2019; Saleem and Byrd, 2021).

In practice, school management can use these findings to design a curriculum that promotes multicultural education in schools. Given that Han students find little meaning in their ethnic identity, schools should teach them to understand their privileged status, the history leading to such racial inequality, and their responsibility to advocate cross-cultural appreciation and acceptance. Ignorance of the meaning of one’s own ethnicity can result in ethnic-racial discrimination and injustice (Moffitt and Rogers, 2022). For Yi students, schools should facilitate their embracement of the meaning of their ethnic identity in relation to mainstream society. Rather than merely fostering a sense of belonging and ethnic pride through cultural festival participation, a thorough understanding of their own ethnic history, hardships, and racial discrimination is necessary to foster self-appreciation and mental health in Yi students in Han-driven school settings (Umana-Taylor et al., 2014; Umaña-Taylor, 2023). These initial programs that will be implemented in Liangshan can be standardized and tailored to other autonomous administrative regions in China, facilitating the advancement of multicultural integration.

Policy wise, a national-level directive is required to foster culturally competent teachers, especially those teaching in classrooms with students of mixed ethnicities. Cultural diversity training for teachers is an indispensable part of implementing multicultural integration policies in schools. The goal is to foster cross-cultural integration and celebrate individual uniqueness, rather than a cultural group feeling that they must assimilate to another.

## Data availability statement

The original contributions presented in the study are included in the article/Supplementary material, further inquiries can be directed to the corresponding author.

## Ethics statement

The studies involving humans were approved by Human Research Ethics Committee, the Hong Kong Polytechnic University. The studies were conducted in accordance with the local legislation and institutional requirements. Written informed consent for participation in this study was provided by the participants' legal guardians/next of kin.

## Author contributions

AL: Conceptualization, Data curation, Formal analysis, Funding acquisition, Investigation, Methodology, Project administration, Resources, Software, Supervision, Validation, Visualization, Writing – original draft, Writing – review & editing. JL: Formal analysis, Methodology, Writing – original draft. HY: Conceptualization, Data curation, Writing – review & editing. ET: Conceptualization, Writing – review & editing. CL: Project administration, Writing – original draft.
